# A behavior change communication intervention, but not livelihood interventions, improves diet diversity and animal-source food consumption among Ghanaian women

**DOI:** 10.29219/fnr.v66.7570

**Published:** 2022-07-27

**Authors:** Elizabeth F. Ludwig-Borycz, Mark L. Wilson, Esi K. Colecraft, Andrew D. Jones

**Affiliations:** 1 Department of Nutritional Sciences, School of Public Health, University of Michigan, Ann Arbor, MI, USA; 2 Department of Epidemiology, School of Public Health, University of Michigan, Ann Arbor, MI, USA; 3 Nutrition and Food Science Department, University of Ghana, Accra, Ghana

**Keywords:** minimum dietary diversity for women, fisheries, value chain intervention, women of reproductive age, microcredit, sub-Saharan Africa, low- and middle-income countries

## Abstract

**Background:**

Women of reproductive age (WRA), especially in sub-Saharan Africa, are vulnerable to micronutrient deficiencies driven largely by poor quality diets. Intervening into food value chains, on which many households in low- and middle-income countries depend for their livelihood, may be a promising approach to improving diets in these contexts.

**Objective:**

In this pilot-scale randomized trial, we evaluated whether a multisectoral, food value chain intervention improved the diet diversity and the consumption of animal-source foods (ASFs) among WRA in Ghana.

**Design:**

Twelve fish-smoking communities in two regions of Ghana with 296 eligible women were randomly assigned to one of three 9-month treatment arms: 1) behavior change communication (BCC) to promote improved diet quality through twice-weekly audio messages and bi-weekly peer-to-peer learning sessions; 2) BCC with microcredit to increase women’s incomes; or 3) BCC with provision of new smoke-oven technology. We assessed baseline-endline and between-treatment arm differences using a 10-food group diet diversity score (DDS), the Minimum Dietary Diversity for Women (MDD-W) indicator, and 7-day frequency of ASF consumption.

**Results:**

Among 118 participants (39 in both treatment arm 1 and treatment arm 3, and 40 in treatment arm 2, with no participant refusals), DDS increased from a mean (SD) of 4.0 (1.3) at baseline to 5.1 (0.9) at endline (*P*-value < 0.0001). The proportion of women achieving the MDD-W indicator nearly doubled from baseline (35.6%) to endline (69.5%) (*P*-value < 0.0001). Frequency of ASF consumption similarly increased for meat and poultry (2.7 (4.1) to 4.7 (5.3); *P*-value < 0.0001) and eggs (1.5 (3.1) to 2.3 (4.9); *P*-value = 0.02). Few differences in these outcomes were observed among treatment arms.

**Conclusions:**

A BCC intervention improved diet diversity and consumption of ASFs among participants. However, neither a group-based microcredit nor improved smoke oven intervention, both of which increased women’s income, led to additional dietary improvements.

## Popular scientific summary

- Women of reproductive age often lack access to healthy diets because of the high costs of micronutrient-rich foods and poor nutritional knowledge.
- A behavior change intervention improved diet diversity and animal-source food consumption among Ghanaian women. Neither a group-based microcredit program nor providing an improved smoke oven enhanced these nutritional outcomes beyond the simple behavior change intervention.
- Acquiring a better understanding of the importance of micronutrient-rich foods for health in part explains the observed dietary improvements.

Women of reproductive age (WRA) in low- and middle-income countries (LMICs) often lack access to healthy diets. In sub-Saharan Africa (SSA), for example, women’s diets are commonly comprised largely of starchy staples and lack the diversity needed to provide appropriate nutrients (1). Low intake of micronutrient-dense foods, especially from animal sources (e.g. meat, poultry, fish, eggs, and dairy), is especially problematic (2–4). Increasing the diet diversity of women, in particular their consumption of animal-source foods (ASFs), has been shown to reduce micronutrient deficiencies among WRA, partly due to the high density and bioavailability of key micronutrients contained within many of these foods (5–7). More diverse diets among women are also associated with lower age-adjusted risk of mortality (4), reduced risk of maternal anemia and adverse pregnancy outcomes (8), and improved nutritional status (9). The low diet diversity observed among many households in SSA may be driven, in part, by low diversity of agricultural production among farming households (10), but is perhaps even more strongly linked to the often high costs of ASFs, thus limiting economic access to such foods (11, 12). Such restricted access may be further exacerbated by inadequate knowledge of the health benefits of such foods (13, 14).

Previous research has highlighted the importance of educating women in LMICs on how to change dietary behaviors to increase diet diversity and ASF consumption. A cluster-randomized intervention in Bangladesh, for example, involving maternal nutrition education to encourage a diverse diet increased the proportion of women consuming five or more food groups per day from ~30% to >80% (15). In Nepal, a quasi-experimental study showed that education of anemic pregnant women in their second trimester was successful at increasing the consumption of ASFS (i.e. red meat, fish, liver, eggs, and dairy) as well as fruits and dark green vegetables (16). In Burkina Faso, another nutritional behavioral change communication intervention along with enhanced homestead food production increased maternal consumption of fruit, meat and poultry, and overall diet diversity (17). Broader evidence reported in reviews of multisectoral interventions promoting improved crop or livestock production also indicates the importance of including nutrition education in such interventions to catalyze improved diets among vulnerable groups (18, 19).

Although women’s knowledge of the benefits of a diverse diet can improve their diets and health, economic access to diverse foods, and ASFs in particular, is also important (13, 20). For low-income households in SSA whose livelihood depends on work in food value chains, whether food production, processing, or retail, intervening through these value chains to improve diets may be especially promising. With regard to ASFs, food value chain interventions may facilitate dietary improvements by promoting the consumption of own-produced animal products, or by stimulating their sale to obtain additional income that could indirectly contribute to more diverse diets (18, 21). For example, a combined agriculture and nutrition intervention in western Kenya increased the diet diversity of children and women who were directly involved in farm diversification activities and improved their nutrition through community-led agricultural activities (22). A similar intervention conducted in South Africa, that combined nutrition education with home gardening, led to improvements in the diet diversity of women (23). In another food production and nutrition education program carried out among households in Bangladesh, Cambodia, Nepal, and the Philippines, both ASF consumption and diet diversity increased, especially among women and children (24). Participating households were also able to invest increased earnings from the sale of vegetables, fruit, poultry, and eggs toward the purchase of more food, which contributed to greater diet diversity (24). Few studies have examined the impact of food value chain interventions on diet diversity and ASF consumption among WRA. Understanding the separate and combined effects of nutrition education and income generation through food value chains is important, given the emerging emphasis on adopting multisectoral food system approaches to promote healthy diets globally. These approaches simultaneously target multiple components of food value chains (e.g. food production, processing, and retail) (25–27). Such knowledge could also help attain Sustainable Development Goals 2 and 3 that target improvements to the health, nutrition, and well-being of women and children (28).

In this study, we evaluated whether a multisectoral, food value chain intervention improved the diet diversity and the consumption of ASFs among WRA in Ghana who smoke fish as their primary livelihood. This pilot-scale, randomized trial was part of a larger formative evaluation that examined the implementation feasibility and plausible impact pathways of reducing anemia through interventions to decrease women fish smokers’ environmental exposures, enhance incomes, increase knowledge about anemia and its determinants, and improve the quality of diets. We hypothesized that enhanced knowledge would increase women’s diet diversity and the consumption of ASFs and further hypothesized that women who received interest-free, rebated microloans for their fish-smoking enterprises, along with corresponding behavior change messages, would demonstrate larger dietary changes as compared with women only receiving behavior change messages. We hypothesized that these larger dietary changes would result from increased purchases of ASFs among women receiving the interest-free, rebated microloans.

## Methods

### Study design and data collection

This study was carried out among 12 fish-smoking communities in the Central and Volta regions of Ghana, representing marine and freshwater fisheries, respectively. We used available government census data on the population and number of households within each region as well as information from key informants on the presence of small-scale fish-smoking enterprises to identify eligible communities. Eligible communities were defined as those located in districts not prone to conflict, in which fish smoking was carried out during 8 or more months of the year, and which had at least 15 households participating in small-scale fish smoking. We selected six communities from each region, ensuring within-region variation in community size and market access. Households in these communities were commonly engaged in wild capture fishing and in activities along the fish value chain as a main economic activity (e.g. smoking fish and selling fresh or processed fish to local and other domestic markets).

Following community selection, we conducted a census in each community, and using the collected census data, we randomly selected 10 households per community that had a woman aged 18–49 years who met the following inclusion criteria: 1) self-reported as not pregnant at the time of enrollment; 2) engaged in small-scale fish smoking as her principal livelihood or had earned income from fish smoking in the past 12 months (‘small-scale’ was defined as an operation with four or fewer functional smoke ovens and no more than three hired laborers); 3) did not plan to move from her community of enrollment for more than 3 weeks during the subsequent 11 months; 4) was willing to accept a no-interest loan for her fishing smoking business and perceived that such a loan would benefit her business; and 5) did not already own an improved ‘Ahotor’-style smoke oven at the time of enrollment. If more than one woman in the same randomly selected household was eligible for the study, we randomly selected just one woman from the household for participation in the study. In total, 296 women across the 12 communities met these inclusion criteria and were considered eligible for participation in the study.

The 12 study communities were subsequently randomly assigned to one of three treatment arms (TAs), such that two communities in both regions participated in each of the three TAs. The interventions were implemented for 9 months in each of the TAs. The first treatment arm (TA1) was an anemia behavior change intervention. Participants received twice-weekly audio messages on provided mobile phones promoting several anemia-mitigating behaviors (e.g. dietary improvements; infection prevention practices; and water, sanitation, and hygiene (WASH) best practices). Participants also joined in bi-weekly, facilitated peer-to-peer learning sessions to reinforce the behavior change practices communicated through the audio messages and to ensure participants listened to all received audio messages. The specific dietary change messages promoted through TA1 included increasing the overall diversity of diets of women to include a broad range of legumes, vegetables, and ASFs as well as special emphasis on increased consumption of ASF (including fish, meat, organ meat, and eggs) to ameliorate deficiencies of iron and key vitamins that underlie nutritional anemia (7, 29). The messages were adapted from educational and health promotion materials used by the Disease Control and Nutrition Departments of the Ghana Health Service as well as the National Malaria Control Programme. Importantly, all study participants, regardless of TA assignment, received these interventions included in TA1. One-third of the participants (*n* = 40 women) received only the TA1 interventions, while other participants in TA2 and TA3 received the TA1 interventions plus the additional interventions described below that were a part of TA2 and TA3, respectively.

Treatment Arm 2 (TA2) participants were additionally provided with assistance aimed at increasing the profitability of their fish-smoking businesses through a group-based microcredit scheme. TA2 participants received two, separate, interest-free loans for their fish-smoking businesses (the second loan included a partial rebate depending on the timing and extent of repayment of the first loan, such that this second loan effectively functioned as a cash transfer). The first loan amount was 1,000 GH¢ (~$US 250 at the time of the study), and the second loan amount was 1,500 GH¢. Participants in TA2 also received entrepreneurship training focused on marketing, book-keeping, and saving strategies for their business as well as twice-monthly text messages conveying market price information for smoked fish.

Participants in TA3 were given an improved ‘Ahotor’-style fish-smoking oven (valued at $US550) to replace the oven that was currently being used, in addition to the TA1 anemia behavior change intervention. The Ahotor oven is designed to reduce emissions from biomass fuel combustion, decrease polycyclic aromatic hydrocarbon levels of smoked fish, and increase fuel efficiency vis-à-vis the traditional Chorkor oven commonly used throughout Ghana. TA3 participants also received training on the use of the Ahotor oven with the aim of reducing harmful respiratory exposures associated with the wood burning and fish-smoking processes.

### Measurement of variables

During the pre-intervention ‘baseline’ period (May–June 2018), a pre-tested, quantitative, household survey instrument was administered by trained enumerators to collect data on household sociodemographic characteristics, women’s fish-smoking practices, empowerment status, and other information. For the index participant, we also collected data from a 24-h dietary recall, with a repeat recall on a nonconsecutive day during the same 7-day period. All these data were again collected from each participating woman at the ‘endline’ after interventions had been completed (May–June 2019). The 24-h diet recalls were collected using standard protocols involving the multiple-pass method and context-specific serving dishes and utensils (30). Data from the second 24-h dietary recalls obtained at both baseline and endline were used to calculate a continuous diet diversity score (DDS) for the baseline and endline periods, respectively. The first dietary recall at baseline was not used due to errors in quantifying specific serving sizes that were corrected in subsequent recalls. The DDS is based on recent consumption (i.e. past 24 h) of foods from 10 different food groups: 1) grains, white roots and tubers, and plantains; 2) pulses (beans, peas, and lentils); 3) nuts and seeds; 4) dairy; 5) meat, poultry, and fish; 6) eggs; 7) dark green leafy vegetables; 8) other vitamin A-rich fruits and vegetables; 9) other vegetables; and 10) other fruits (7). The consumption of each food group was coded as 1 if any amount of that food was consumed in the past 24 h, and as 0 otherwise. Based on this, the Minimum Dietary Diversity for Women (MDD-W) indicator was created by summing the values for each of the 10 food groups. The MDD-W indicator, ranging from 0 to 10, served as a proxy for the micronutrient adequacy of diets of WRA (7). Women who ate five or more of the 10 food groups were considered to have met the MDD-W indicator (7).

ASF consumption was measured from each participant’s self-reported frequency of consumption of any ASF in the past 7 days and used to create variables representing different groups of ASF consumed. Meat and poultry consumption, for example, was based on the number of days and average number of times per day items from each of nine categories of meat and poultry were consumed. This same approach was used to assess fish consumption (based on eight categories of fish and shellfish), egg consumption (based on two categories), and dairy consumption (based on five categories). To complement these ASF consumption frequency data, we also assessed household expenditures (GH¢) ASF items in the past 7 days for the same ASF categories based on respondent recall of expenditures on each food item.

Recent household income was assessed based on a self-reported questionnaire documenting all income-generating activities by all household members in the past 30 days. Additional earnings from land sales or other sources (e.g. remittances and social welfare benefits) were added to calculate the total earnings per household.

Characteristics of the index woman in each household included self-reported age and education level. At baseline and endline, we further assessed participants’ knowledge of the dietary causes of anemia and strategies to prevent anemia through dietary change. These knowledge assessments were aimed at understanding participants’ uptake of the mobile phone audio messages focused on dietary change that were shared as part of the TA1 intervention. For each knowledge question pertaining to diet and anemia, we coded respondents’ answers as ‘correct’ or ‘incorrect’ based on a pre-specified answer key. For all questions, there were multiple correct responses. If the respondent provided at least one correct response for the question, it was coded as ‘correct’.

### Statistical analysis

Differences between baseline and endline values were evaluated by treatment group for DDS, recent consumption of specific foods group, and frequency of consumption of ASFs in the past 7 days. For continuous variables (i.e. DDS, ASF consumption meat and poultry, ASF consumption fish, ASF consumption eggs, and ASF consumption milk and dairy), the Wilcoxon signed-rank test for paired data was used for non-normally distributed data, and ANOVA was used for normally distributed data. To assess differences in categorical variables (i.e. MDD-W achieved and each food category that comprises the DDS), Pearson’s chi-squared test, McNemar’s test for correlated data, and Fisher’s exact test were used. Differences in DDS, MDD-W, and frequency of ASF consumption across treatment groups at baseline and endline, respectively, were assessed using ANOVA for continuous variables and Pearson’s chi-squared test for categorical variables.

We further analyzed the association of treatment arm with DDS and ASF consumption at study endline using ordinary least squares linear regression models, and of treatment arm with MDD-W using logistic regression models. For each set of analyses, three models were constructed, adjusting for: 1) no covariates, 2) the baseline value of the dependent variable, and 3) the baseline value of the dependent variable as well as baseline household income and maternal education status.

Statistical analysis was carried out in SAS version 9.4 (Cary, NC). We report statistical significance at the *P* < 0.05 and *P* < 0.01 levels as well as at the *P* < 0.1 level to indicate potentially meaningful trends in this pilot-scale study.

### Ethical approval

The study protocol was approved by the University of Michigan Health Sciences and Behavioral Sciences Institutional Review Board and the Ethics Committee for Basic and Applied Sciences at the University of Ghana, Legon. Comprehensive informed consent was obtained from all study participants. The trial is registered at clinicaltrials.gov (NCT03498755) (31).

## Results

Overall, 118 (98.3%) women were included in analyses (39 in TAs 1 and 3 and 40 in TA2). Two women who relocated to different communities following the baseline assessment were lost to follow-up. No women refused to participate in the study when invited at baseline. The mean (SD) age of participants at baseline was 39.2 (6.6) years, and average monthly household income was GH¢250 (607) (~US$46) (Supplemental Table 1). More than half of the women (55.1%) had never attended school. Mean household size was 7.1 (3.0), and the mean number of under-18 children in households was 3.6 (2.0) (Supplemental Table 1).

### Intervention impacts on diet diversity

DDS among participants increased from an average of 4.0 (1.3) at baseline to 5.1 (0.9) at endline (*P*-value < 0.0001) (Table 1). We observed no differences in DDS across treatment arms at baseline (TA1: 4.0 (1.3); TA2: 4.0 (1.5); TA3: 3.9 (1.1); *P*-value = 0.94) or at endline (TA1: 5.3 (0.8); TA2: 4.9 (0.9); TA3: 5.2 (1.0); *P*-value = 0.20).

**Table 1:** Diet diversity and frequency of animal-source food consumption among study participants, by treatment arm and data collection period

	Combined treatment arms (*n* = 118)		Treatment arm 1 (*n* = 39)		Treatment arm 2 (*n* = 40)		Treatment arm 3 (*n* = 39)	
	Baseline	Endline	Baseline	Endline	Baseline	Endline	Baseline	Endline
	Mean (SD) or %							
Diet diversity								
Diet diversity score (DDS)	4.0 (1.3)	5.1 (0.9)***	4.0 (1.3)	5.3 (0.8)***	4.0 (1.5)	4.9 (0.9)***	3.9 (1.1)	5.2 (1.0)***
%Achieving MDD-W indicator	35.6	69.5***	33.3	79.5***	40.0	60.0*	33.3	69.2***
Any recent (24 h) consumption of…, %								
Grains, roots, and tubers	94.1	100***	92.3	100*	95.0	100	94.9	100
Pulses	6.8	21.2***	10.3	18.0	2.5	25.0***	7.7	20.5*
Nuts and seeds	15.3	21.2	10.3	28.2**	20.0	12.5	15.4	23.1
Dairy	14.4	17.8	7.7	15.4	25.0	12.5*	10.3	25.6*
Meat, poultry, and fish	83.1	100***	87.2	100**	82.5	100***	79.5	100***
Eggs	13.6	25.4**	15.4	28.2	10.0	17.5	15.4	30.8*
Dark green leafy vegetables	77.1	100***	84.6	100**	67.5	100***	79.5	100***
Other vitamin A-rich fruits and vegetables	6.8	15.3**	7.7	23.1*	10.0	15.0	2.6	7.7
Other vegetables	82.2	100***	82.1	100***	77.5	100***	87.2	100**
Other fruits	5.1	10.2	2.6	12.8	12.5	7.5	0	10.3**
Frequency of ASF consumption (times consumed in past 7 days)								
Meat and poultry	2.7 (4.1)	4.7 (5.3)***	2.6 (4.6)	4.9 (5.1)***	2.7 (3.9)	5.3 (6.5)**	2.7 (3.8)	4.0 (4.2)**
Fish	18.2 (13.2)	17.9 (14.8)	16.7 (9.2)	18.4 (10.6)	20.6 (18.9)	21.0 (21.3)	17.2 (8.5)	14.4 (8.5)
Eggs	1.5 (3.1)	2.3 (4.9)**	1.7 (4.2)	1.5 (1.9)	1.6 (2.9)	3.0 (5.9)*	1.8 (1.8)	2.6 (5.9)
Dairy	2.3 (6.0)	1.6 (2.7)	2.4 (6.8)	1.2 (2.5)	2.4 (5.1)	2.3 (3.3)	2.3 (6.0)	1.2 (2.0)

Values are means (SD) or percentages. The Wilcoxon signed-rank test was used to test for differences among non-normally distributed continuous variables. McNemar’s test for correlated data and Fisher’s exact (for cell counts less than 5) test were used to test for differences among dichotomous variables. Statistical significance is shown for differences in characteristics between baseline and endline by treatment group and for all groups combined.

* *P* < 0.1;

** *P* < 0.05;

*** *P* < 0.01.

Abbreviations: ASF: animal-source food; DDS: diet diversity score; MDD-W: minimum dietary diversity for women.

In linear regression models (Table 2), unadjusted (Model 1) analyses showed that DDS was lower at endline among TA2 participants compared with TA1 participants (β coefficient: -0.36; *P*-value = 0.09). After adjusting for baseline DDS (Model 2), this difference persisted (β coefficient: -0.36; *P*-value = 0.09), yet further adjustment for baseline household income and maternal education status (Model 3) weakened the association (β coefficient: -0.31; *P*-value = 0.14).

**Table 2:** Regression analyses examining the association between treatment arm (TA) and diet diversity at study endline

Dependent variable	Model 1	Model 2	Model 3
Diet diversity score (DDS)			
TA1 (reference)	–	–	–
TA2	-0.36*	-0.36*	-0.31
TA3	-0.08	-0.07	-0.07
Minimum diet diversity for women (MDD-W)			
TA1 (reference)	–	–	–
TA2	0.39 (0.14, 1.05)	0.37* (0.13, 1.01)	0.38 (0.13, 1.08)
TA3	0.58 (0.21, 1.63)	0.58 (0.20, 1.63)	0.55 (0.19, 1.61)

Values for models using endline DDS as the dependent variable are β coefficients from ordinary least squares regression models. Values for models using endline MDD-W as the dependent variable are OR (95% CI) from logistic regression models. In Model 1, no covariates were included in the models. In Model 2, regressions were adjusted for the baseline value of the dependent variable. In Model 3, regressions were adjusted for the baseline value of the dependent variable as well as baseline household income and maternal education status.

* *P* < 0.1;

** *P* < 0.05;

*** *P* < 0.01.

Abbreviations: DDS: diet diversity score; MDD-W: minimum dietary diversity for women; ref: reference group for regression analyses.

Recent consumption of all foods that comprised the DDS increased between baseline and endline (Table 1). However, increased DDS from baseline to endline was especially driven by greater consumption of meat, poultry, and fish (83.1–100%); dark green leafy vegetables (77.1–100%); other vegetables (82.2–100%); to a lesser extent by pulses (6.8–21.2%); eggs (13.6–25.4%); and other vitamin A-rich fruits and vegetables (6.8–15.3%) (Table 1). The greatest increases occurred for the consumption of other vitamin A-rich fruits and vegetables in TA1 (7.7–23.1%), eggs in TA2 (2.5–25.0%), and pulses in TA3 (15.4–30.8%).

The proportion of women achieving the MDD-W indicator (i.e. consuming ≥5 food groups) nearly doubled from baseline (35.6%) to endline (69.5%) (*P*-value < 0.0001) (Table 1). We observed no statistical differences among the three TAs, at either baseline or endline, in the proportion of women achieving the MDD-W. The largest percentage of women achieving the MDD-W indicator at endline was in TA1 (79.5%), followed by TA3 (69.2%) and TA2 (60.0%) (*P*-value = 0.17 for differences in proportions at endline). Unadjusted and adjusted regression analyses confirmed that women in TA2 and TA3 had lower odds of achieving the MDD-W indicator as compared to TA1 participants, but none of these differences were statistically significant (Table 2).

### Intervention impacts on consumption of ASFs

ASF consumption did not differ at baseline among treatment arms for meat and poultry (*P*-value = 0.59), fish (*P*-value = 0.77), eggs (*P*-value = 0.84), or dairy (*P*-value = 0.99). Among all study participants, the frequency of ASF consumption in the past 7 days increased from baseline to endline for meat and poultry (2.7 (4.1) to 4.7 (5.3); *P*-value < 0.0001) as well as for eggs (1.5 (3.1) to 2.3 (4.9); *P*-value = 0.02), but no change in consumption of fish or dairy was observed (Table 1). Frequency of fish consumption at baseline (2–3 times per day) was already higher as compared to other ASFs (2–3 times per week) (Table 1). Changes in the frequency of consumption of meat and poultry were driven primarily by increased consumption of chicken, beef, pork, and to a lesser degree goat (Supplementary Table 2). Comparisons within treatment arms showed that meat and poultry consumption consistently increased from baseline to endline (TA1: 2.6 (4.6) to 4.9 (5.1), *P*-value = 0.005; TA2: 2.7 (3.9) to 5.3 (6.5), *P*-value = 0.02; TA3: 2.7 (3.8) to 4.0 (4.2), *P*-value = 0.02), and egg consumption nearly doubled among TA2 participants (Table 1). We observed no statistically significant differences among treatment arms in the frequency of consumption of any of the ASFs at endline. However, frequency of consumption of all ASFs at endline was highest among TA2 participants as compared to other treatment arms (Table 1).

Unadjusted and adjusted regression analyses confirmed these overall trends (Table 3), indicating greater consumption of ASFs among TA2 participants as compared to TA1 participants; however, the only statistically significant difference was more frequent dairy consumption among TA2 participants as compared to TA1 participants.

**Table 3:** Regression analyses examining the association between treatment arm (TA) and frequency of recent animal-source food consumption at study endline

Dependent variable	Model 1	Model 2	Model 3
Frequency of meat and poultry consumption			
TA1 (reference)	–	–	–
TA2	0.40	0.38	0.39
TA3	-0.87	-0.89	-1.05
Frequency of fish consumption			
TA1 (reference)	–	–	–
TA2	2.59	2.39	1.61
TA3	-3.92	-3.95	-4.30
Frequency of egg consumption			
TA1 (reference)	–	–	–
TA2	1.46	1.48	1.52
TA3	1.10	1.23	1.33
Frequency of dairy consumption			
TA1 (reference)	–	–	–
TA2	1.05*	1.05*	0.76
TA3	0.03	0.04	0.11

Dependent variables in all models are the number of times the animal-source food indicated was consumed by the index respondent in the past 7 days. β coefficients from ordinary least squares regression models are shown. In Model 1, no covariates were included in the models. In Model 2, regressions were adjusted for the baseline value of the dependent variable. In Model 3, regressions were adjusted for the baseline value of the dependent variable as well as baseline household income and maternal education status.

* *P* < 0.1;

**P < 0.05;

***P < 0.01.

### Intervention impacts on knowledge of the causes of anemia and prevention strategies

The proportion of women who correctly identified causes of anemia and prevention strategies increased from baseline to endline for all treatment arms (Table 4). Similarly, knowledge of foods that protect against anemia increased from baseline to endline across all treatment arms, although >80% of participants already demonstrated this knowledge at baseline. However, knowledge of the superiority of iron from ASFs, and of iron-rich foods for women as compared to men, was low at baseline and increased substantially for all treatment arms by endline (Table 4).

**Table 4:** Knowledge of the causes of anemia and prevention strategies by treatment arm (TA) at study baseline and endline

Knowledge assessment question	TA1 (*n* = 39)			TA2 (*n* = 40)			TA3 (*n* = 39)		
	Baseline (%)	Endline (%)	*Х* ^2^	Baseline (%)	Endline (%)	*Х* ^2^	Baseline (%)	Endline (%)	*Х* ^2^
1. What causes anemia?	48.7	92.3	15.2***	55.0	97.5	17.0***	59.0	100	16.0***
2. How do you think that anemia can be prevented?	43.6	84.6	10.7***	57.5	92.5	12.3***	59.0	100	16.0***
3. Which foods protect against anemia?	89.7	97.4	1.8	92.5	100	3.0*	84.6	100	6.0**
4. Why is iron from animal-source foods superior to iron from plant foods?	20.5	59.0	10.7***	35.0	70.0	8.9***	25.6	69.2	15.2***
5. Why do women need more iron-rich foods to prevent anemia than men?	56.4	84.6	8.1***	70.0	97.5	9.3***	51.3	100	19.0***

Values are proportions indicating the percentage of participants that responded correctly to each question at each time point. Differences in the proportion of respondents correctly answering the questions between baseline and endline were assessed using the McNemar’s test for correlated data.

* *P* < 0.1;

** *P* < 0.05;

*** *P* < 0.01.

Correct answers for Question 1 included: poor quality diet; malaria; worm infestation; exposure to smoke; pregnancy. Correct answers for Question 2 included: sleeping under a treated mosquito bed net; reducing exposure to smoke; observing personal hygiene; observing a clean environment; ensuring food is well cooked before eating. Correct answers for Question 3 included: small fish eaten whole; other kinds of fish; beef; duck; chicken; pork; bush meat; offals of animals; dark green leafy vegetables; beans; nuts; seeds. Correct answers for Question 4 included: superior quality iron; animal foods have iron that can be better absorbed. Correct answers for Question 5 included: women lose blood through menstruation; pregnancy and childbirth deplete iron stores; breastfeeding increases women’s requirement for iron; women’s low social status limits their intake of nutrient-rich foods.

### Intervention impacts on ASF expenditures

For all treatment arms combined, household expenditures on chicken, beef, goat, and eggs increased from baseline to endline, with chicken and goat expenditures showing the largest increases (Table 5). Increased ASF expenditures were consistently observed in all treatment arms, with participants in TA2 showing the largest increases in expenditures on beef, while participants in TA3 showed the largest increases in chicken and eggs. Household expenditures on fish were larger by far than any other ASF category, though no statistically significant changes in fish expenditures were observed across any of the treatment arms between baseline and endline.

**Table 5:** Household expenditures on animal-source foods, by treatment arm at study baseline and endline

Household expenditures in past 7 days on	All treatment arms (*n* = 118)		Treatment arm 1 (*n* = 39)		Treatment arm 2 (*n* = 40)		Treatment arm 3 (*n* = 39)	
	Baseline Mean (SD)	Endline Mean (SD)	Baseline Mean (SD)	Endline Mean (SD)	Baseline Mean (SD)	Endline Mean (SD)	Baseline Mean (SD)	Endline Mean (SD)
Pork	0.3 (1.6)	0.8 (3.5)	0.3 (1.6)	2.1 (5.9)*	0.6 (2.2)	0.1 (0.5)	0	0.1 (0.8)
Beef	3.1 (6.3)	5.0 (7.7)***	2.6 (7.7)	4.3 (6.3)	4.2 (6.7)	6.7 (8.1)*	2.4 (4.1)	4.0 (8.4)
Corned beef	0	0.3 (2.8)	0	0	0	0	0	0.9 (4.8)
Goat	0.7 (3.9)	5.3 (27.8)**	0.9 (4.3)	0.9 (3.5)	0.8 (4.7)	1.1 (4.0)	0.4 (2.4)	14.1 (47.3)**
Mutton	0.1 (1.4)	0.1 (0.9)	0.4 (2.4)	0	0	0	0	0.3 (1.6)
Bushmeat/Wild Game/Game birds	1.0 (5.5)	0.9 (6.1)	1.8 (7.9)	2.8 (10.5)	0	0	1.3 (5.3)	0
Other meat (dog, cat, etc.)	0.3 (2.8)	0.4 (4.6)	0	1.3 (8.0)	0.8 (4.7)	0	0	0
Chicken	6.8 (14.8)	12.2 (20.8)***	8.6 (21.7)	13.5 (23.4)	8.5 (12.0)	9.5 (9.7)	3.3 (6.3)	13.6 (25.9)***
Other poultry	0.0 (0.5)	0	0.1 (0.8)	0	0	0	0	0
Fish	55.7 (59.2)	49.3 (61.7)	65.9 (85.8)	53.9 (78.5)	54.1 (45.0)	53.8 (62.6)	47.2 (34.1)	40.1 (37.3)
Milk	2.7 (5.4)	3.0 (5.6)	3.0 (6.3)	3.9 (8.4)	3.2 (6.2)	3.0 (3.7)	1.9 (2.9)	1.9 (2.8)
Eggs	1.7 (3.0)	2.2 (2.7)***	1.3 (2.0)	2.0 (3.2)	1.9 (3.3)	2.1 (2.0)	1.8 (3.4)	2.5 (2.8)*

Values are mean (SD) household expenditures (GH¢) on the specific food categories shown over the past 7 days. Means are based on expenditures across the entire sample, including household that did not purchase the food item in the previous 7 days. The Wilcoxon signed-rank test was used to test for differences among non-normally distributed continuous variables. Statistical significance is shown for differences in characteristics between baseline and endline by treatment group and for all groups combined.

* *P* < 0.1;

** *P* < 0.05;

*** *P* < 0.01.

## Discussion

The results of this study demonstrated that a behavior change intervention, combining peer-to-peer learning sessions with mobile phone audio messages promoting diverse anemia-mitigating behaviors, improved diet diversity and consumption of ASFs among Ghanaian WRA. Neither a group-based microcredit intervention that incorporated entrepreneurship training and provision of market price information, nor an intervention that provided participants with an improved smoke oven and training on its use, additionally improved diet diversity or consumption of ASFs beyond that of the behavior change intervention alone.

Across all participants, DDS improved by an average of more than one point (or 27.5%) during the 9-month intervention, and the proportion of women achieving the MDD-W indicator nearly doubled from 35.6% at baseline to 69.5% at the end of the intervention. These changes in diet diversity were driven largely by greater consumption of meat and poultry, dark green leafy vegetables, and other vegetables. The frequency of consumption of meat and poultry, as well as eggs, also increased markedly between baseline and endline. We observed few differences among treatment arms in these dietary changes.

While no studies that we are aware of have intervened into fisheries value chains in the same manner as our study, previous multisectoral interventions targeting both agricultural improvements and dietary behavior change have observed similar increases in diet diversity and ASF consumption among study participants as we observed in our study. These studies have intervened to diversify or enhance homestead food production, while also providing nutrition education. For example, a combined nutrition education and home gardening intervention conducted among South African women led to a one-point increase in a DDS (based on nine food groups) from baseline to endline mainly driven by increases in consumption of meat, poultry, and fish; dairy; and legumes and nuts (23). A similar study in western Kenya that combined nutrition education with efforts to diversify farms (i.e. through poultry rearing and kitchen gardens) observed a nearly one-point increase in diet diversity among children aged 12-23 months (22). However, the designs of those two studies precluded differentiating between individual effects of the nutrition education and agriculture interventions. Another study in Cambodia that used farmer field and business schools combined with nutrition education to improve farmers’ linkages to markets as well as child diets also observed a nearly one-point increase in a DDS among preschool-aged children whose households received the combined interventions (32). The diet diversity of children in households that received farm market training only also increased, but to a lesser extent than that of children whose households received the combined intervention. Finally, in Ghana, a clustered randomized controlled trial of an intervention combining home poultry rearing and gardening with nutrition education showed that preschool-aged children in the intervention group were more likely to meet a minimum standard for a diverse diet (80.2% of intervention children vs. 69.5% of control children), and to have consumed eggs in the previous day (31.5% of intervention children vs. 22.6% of control children) (33). Because this study compared the combined interventions to a control group, the investigators were not able to assess the independent effect of nutrition education.

Though the interventions in our study differed among the three TAs, we observed few statistically significant differences in the magnitude of TA-specific changes in diet diversity. This was true despite large overall differences in some outcomes between TAs. For example, at endline, 60% of TA2 participants met the MDD-W indicator, while this proportion was nearly 20 percentage points higher among TA1 participants (79.5%). Furthermore, the frequency of consumption of all ASFs at endline was highest among TA2 participants as compared to other TAs. The small number of participants may have contributed to these limited statistically significant differences in outcomes by TAs. However, the study design also contributed to these findings. All TAs received the interventions provided to TA1. This design was appropriate for the goals of the larger formative evaluation that was being carried out but minimized exposure differences among TAs.

The improved diet diversity that was observed is consistent with our hypothesized program impact pathway that in part focused on enhanced knowledge of the importance of diet diversity and ASF consumption for women’s health and well-being. We did not condition on diet knowledge in our analyses because we hypothesized improved diet knowledge to be on the causal pathway between our interventions and the diet outcomes we assessed. The TA1 behavior change curriculum that women from all TAs participated emphasized the health benefits of diet diversity, generally, and specifically the protective properties of certain foods for preventing and treating anemia, especially ASFs and micronutrient-rich plants. Consistent with this hypothesis, we observed that the proportion of women who correctly identified the causes of anemia and prevention strategies increased across all TAs during the intervention, as did knowledge of the importance of iron from ASFs, and of iron-rich foods for women.

Another hypothesis was that women in TA2, who received interest-free, rebated microloans for their fish-smoking enterprises, would demonstrate larger dietary changes compared with women in TA1 or TA3, given their access to additional income (from increased business profits) that could be used to purchase more ASFs and more diverse foods. Previous studies that have provided cash transfers to households have demonstrated that this intervention leads to larger changes in diet-related behaviors compared to control conditions (34–38). This is especially true when combined with behavior change communication (BCC). For example, a randomized controlled trial in Bangladesh testing the effectiveness of a nutrition-sensitive social protection intervention on consumption of a multiple-micronutrient powder among children 6–59 months found that children whose households received a cash transfer and BCC were 32.0 percentage points more likely to have ever consumed the micronutrient powder over the 2 year intervention period relative to the control group that received no intervention (39). Households that received a cash transfer only were just 12.6 percentage point more likely to have ever consumed the powder (39). Similarly, in Nepal, a cluster-randomized controlled trial found that pregnant women receiving an unconditional cash transfer combined with participatory health education consumed 0.4 more food groups in the previous day than control women (40). Furthermore, cash transfers can benefit health and wellness outside of diet-related behaviors alone, extending to the accumulation of productive assets, non-farm enterprises, small livestock ownership, flexibility of labor allocation in the home, and reduced child labor (37). However, cash transfers appear not to be effective in all contexts. In Mexico, the evaluation of a national food support program found that diet diversity of children aged 6–23 months in households receiving a cash transfer with education did not improve relative to a control group (41). Evidence from our study does not support the hypothesis that TA2 participants (who received rebated, interest-free loans) or TA3 participants (who could earn additional income from more efficient smoke ovens) experienced larger dietary changes than TA1 participants. Despite the substantial potential of TA2 and TA3 women to benefit financially from such interventions (as evidenced by the low monthly household incomes observed) and the effectiveness of the interventions at generating increased income (as described below), TA2 and TA3 women did not show additional dietary improvements. DDS and MDD-W did increase among TA2 participants, but the absolute change in the mean DDS and prevalence of women achieving the MDD-W were lower among TA2 participants than TA1 and TA3 participants. However, these two indicators of diet diversity are based on a single 24-h recall, which likely misses important variation in usual diet among participants. For example, TA2 participants demonstrated the largest increase in consumption of meat/poultry and eggs compared with the other TAs, as well as the greatest increase in household expenditures on beef. These findings align with other data from our study (not shown) that indicate total earnings from fish smoking among TA2 participants were nearly triple those of participants in the other TAs, although earnings did increase among TA3 participants as well, which may have contributed to the higher ASF expenditures (on chicken, eggs, and goat meat) among TA3 participants (42). Therefore, higher income used to purchase ASFs may have contributed to the changes in ASF consumption observed. However, given the variability in changes observed across all TAs, this was not the only contributing factor, and our data do not allow us to establish whether income earned from fish smoking or other specific sources contributed to changes in ASF expenditures.

The small number of participants in this pilot study, which was part of a larger formative evaluation examining the implementation feasibility of the described interventions, may have restricted our ability to detect certain potential statistically significant differences among TAs. Furthermore, while our study design was appropriate for the goals of the formative evaluation being undertaken, the absence of a control group that received no intervention limited our ability to differentiate impacts by treatment arm because TA2 and TA3 also received the behavior change interventions of TA1. The lack of a pure control group also suggests that the observed changes in diet diversity and ASF consumption could be due to secular trends in diets that occurred between baseline and endline. A single, 24-h diet recall was used to construct the DDS and MDD-W indicators, yet these indicators do not capture intra-individual differences in diet that are important for understanding usual dietary patterns. However, our assessment of 7-day ASF consumption frequency complemented these 24-h diet indicators and contributed to a more comprehensive assessment of participants’ diets. Finally, although seasonality may contribute to dietary differences of women in these two regions of Ghana, our assessment of outcomes at baseline and endline occurred during the same time of year (May–June).

Among the strengths of our study was that women enthusiastically participated in the interventions, with only two lost to follow-up. Participants demonstrated a high uptake of the behavior change messages (82.5% of messages sent were listened to by participants), high participation in educational activities (73% of all TA1 peer-to-peer learning sessions were attended, and 86% of all TA3-specific trainings were attended by TA3 participants), high loan repayment (100 and 97.5% of TA2 participants repaid their loans during the first and second loan cycles, respectively), and comprehensive uptake of the new Ahotor ovens (all TA3 participants consented with no objections to replacing their old Chorkor oven with a new Ahotor oven). In addition, the selection of participants was designed to be representative of the two fishing regions, with eligible fish-smoking communities identified using government census data and key informant insights to ensure within-region variation in community size and market access. Furthermore, we used a pre-tested household survey instrument that was administered in the language of the participant.

In conclusion, a 9-month behavior change intervention, which was focused on anemia mitigation, improved diet diversity and the consumption of ASFs among Ghanaian WRA. Neither a group-based microcredit intervention nor providing participants with an efficient oven (both intended in part to increase income) improved diet diversity or the consumption of ASFs more than the behavior change intervention alone. It is likely that enhanced knowledge partly explains the observed dietary changes because knowledge and understanding of the importance of micronutrient-rich foods for health increased among participants in all three TAs. Fish consumption was already high at baseline and did not change with the interventions, suggesting that more frequent fish consumption should likely not be emphasized in future programs to improve the micronutrient adequacy of diets in this population. However, programs aimed at enhancing the quality of fish consumed (e.g. small fish, consumed whole) may be quite relevant. Larger-scale studies are needed to better understand the dietary impacts of interventions into fisheries value chains, and to further elucidate the mechanisms of such impacts.

## Conflict of interest and funding

The authors declare no conflict of interest. This research was supported with a grant from the Bill & Melinda Gates Foundation (OPP1182940).

## Author contributions

ELB analyzed the data and wrote the first draft of the manuscript; ADJ cowrote the manuscript. MLW and EKC critically revised the manuscript. ELB and ADJ had primary responsibility for the final content of the manuscript. All authors read and approved the final manuscript. No author has any conflict of interest financially or otherwise in the production of this manuscript.

## Supplementary Material

A behavior change communication intervention, but not livelihood interventions, improves diet diversity and animal-source food consumption among Ghanaian women
