# Supplementary material for: A behavior change communication intervention, but not livelihood interventions, improves diet diversity and animal-source food consumption among Ghanaian women
Source: Food Nutr Res. 2022 Jul 27;66:10.29219/fnr.v66.7570. doi: 10.29219/fnr.v66.7570 (PMC9338446; doi:10.29219/fnr.v66.7570)
Supplement: A behavior change communication intervention, but not livelihood interventions, improves diet diversity and animal-source food consumption among Ghanaian women [file FNR-66-7570-s001.docx]

|  | Combined Treatment Arms  (n=118) | Treatment Arm 1  (n=39) | Treatment Arm 2  (n=40) | Treatment Arm 3  (n=39) | $X^{2}$ |
| --- | --- | --- | --- | --- | --- |
|  | Mean (SD) or % | | | | |
| Age, years | 39.2 (6.6) | 41.3 (6.2) | 39.4 (6.2) | 36.8 (6.7) | 0.009*** |
| Region |  |  |  |  | 0.97 |
| Central | 50.00 | 48.7 | 50.0 | 51.28 |  |
| Volta | 50.00 | 51.3 | 50.0 | 48.72 |  |
| Education level of index women |  |  |  |  | 0.88 |
| Don’t Know | 2.54 | 2.6 | 2.5 | 2.6 |  |
| None | 29.66 | 33.3 | 22.5 | 33.33 |  |
| Nursery | 25.42 | 18.0 | 32.5 | 25.6 |  |
| Primary School | 13.56 | 15.4 | 10.0 | 15.4 |  |
| Junior High School | 22.03 | 23.1 | 22.5 | 20.5 |  |
| Senior High School | 6.78 | 7.7 | 10.0 | 2.6 |  |
| Household income (past month), GH¢ | 250 (607) | 194 (554) | 322 (653) | 233 (618) | 0.27 |
| Household size (total members) | 7.1 (3.0) | 7.1 (3.1) | 6.9 (2.5) | 7.3 (3.3) | 0.87 |
| Under-18 children in household | 3.6 (2.0) | 3.6 (2.3) | 3.6 (1.8) | 3.7 (1.9) | 0.93 |

**Supplemental Table S1**. Characteristics of study participants, by treatment arm at study baseline.

Values are mean (SD) or percentages. Pearson’s chi-squared and Fisher’s exact (for cell counts less than 5) tests were used to test statistical differences for categorical variables between treatment arms at baseline. The Wilcoxon signed-rank test for paired data and ANOVA were used to test statistical differences between treatment arms at baseline for non-normally and normally distributed continuous variables, respectively; **P*<0.1; ***P*<0.5; ****P*<0.01.

**Supplemental Table S2**. Frequency of recent consumption of meat and poultry by the index participant, by treatment arm at baseline and endline.

|  | Combined Treatment Arms  (n=118) | | Treatment Arm 1  (n=39) | | Treatment Arm 2  (n=40) | | Treatment Arm 3  (n=39) | |
| --- | --- | --- | --- | --- | --- | --- | --- | --- |
|  | Baseline | Endline | Baseline | Endline | Baseline | Endline | Baseline | Endline |
|  | Mean (SD) | | | | | | | |
| Frequency of consumption of…in past seven days |  |  |  |  |  |  |  |  |
| Pork | 0.0 (0.2) | 0.3 (1.6)** | 0.0 (0.2) | 0.8 (2.7)** | 0.1 (0.3) | 0.1 (0.3) | 0.0 (0.0) | 0.1 (0.3) |
| Beef | 1.2 (2.5) | 1.9 (3.4)** | 1.1 (2.4) | 1.8 (3.0)* | 1.2 (2.5) | 3.0 (4.7)*** | 1.5 (2.5) | 0.9 (1.6) |
| Corned beef | 0.0 (0.0) | 0.1 (0.6) | 0 | 0.0 (0.0) | 0 | 0 | 0.0 (0.0) | 0.2 (1.0) |
| Goat | 0.1 (0.7) | 0.3 (0.9)* | 0.3 (1.1) | 0.1 (0.4) | 0.1 (0.5) | 0.2 (0.7) | 0.0 (0.2) | 0.5 (1.3)** |
| Mutton | 0.0 (0.2) | 0.0 (0.2) | 0.1 (0.3) | 0 | 0 | 0 | 0.0 (0.0) | 0.1 (0.3) |
| Bushmeat/Wild game | 0.2 (0.9) | 0.0 (0.2) | 0.2 (1.0) | 0.1 (0.4) | 0 | 0 | 0.3 (1.1) | 0.0 (0.0) |
| Other meat (dog, cat, etc.) | 0.0 (0.1) | 0.0 (0.1) | 0 | 0.0 (0.2) | 0.0 (0.2) | 0 | 0.0 (0.0) | 0.0 (0.0) |
| Chicken | 1.1 (1.9) | 2.1 (2.9)*** | 0.9 (1.7) | 2.0 (3.0)** | 1.4 (2.0) | 2.1 (2.7) | 0.9 (1.9) | 2.3 (3.0)** |
| Other poultry | 0.0 (0.2) | 0 | 0.1 (0.3) | 0 | 0 | 0 | 0.0 (0.0) | 0.0 (0.0) |

Values are mean (SD) number of times each food group shown was consumed by a household member in the past seven days. The Wilcoxon signed-rank test was used to test for differences between baseline and endline by treatment group, and for all groups combined. Statistical significance is shown for differences in characteristics between baseline and endline by treatment group and for all groups combined. **P*<0.1; ***P*<0.05; ****P*<0.01.
